# A Case Report and Brief Literature Review on Dedifferentiated Chondrosarcoma in Proximal Phalanx: A Rare Location

**DOI:** 10.7759/cureus.29105

**Published:** 2022-09-13

**Authors:** Ketav Desai, Shiguang Liu, Brett Baskovich, Raafat Makary

**Affiliations:** 1 Pathology, University of Florida College of Medicine – Jacksonville, Jacksonville, USA; 2 Pathology and Laboratory Medicine, University of Florida College of Medicine – Jacksonville, Jacksonville, USA; 3 Pathology, Mount Sinai Hospital, New York, USA; 4 Pathology, University of Florida, Jacksonville, USA

**Keywords:** tert promoter, idh, sarcoma, dedifferentiated chondrosarcoma, chondrosarcoma of hand

## Abstract

Dedifferentiated chondrosarcoma (DDCS) is a rare entity, constituting only 1-2% of all primary bone tumors, and has a dismal prognosis. Nearly two-thirds of the primary tumors of DDCSs are found in the appendicular skeleton, mostly involving the femur, humerus, and pelvis. DDCS of the small bones of the hand and foot are exceedingly rare with only four cases documented in the literature so far. In this report, we present a case of a 91-year-old woman with a rapidly growing bone tumor initially thought to be a trigger finger, which, on histologic examination of the amputation, turned out to be DDCS. On a follow-up CT scan, multiple pulmonary metastases were identified. Next-generation sequencing identified isocitrate dehydrogenase 2 (*IDH2*) (p.R172S, c.516G>T), *TERT* (c.-146C>T), and *TP53* (c.559+1G>A) mutations. Microsatellite instability was equivocal and tumor mutation burden was low. Due to the advanced age of the patient, she was given palliative treatment and was alive at the six-month follow-up.

## Introduction

Chondrosarcoma is the second most common primary sarcoma of the bone overall. However, it is the most common primary sarcoma of the bone in patients over 50 years of age [1]. The term dedifferentiated chondrosarcoma (DDCS) was introduced by Dahlin and Beabout in 1971 and is applied to a high-grade sarcoma present next to a low-grade malignant cartilage-forming tumor [2]. The high-grade sarcomatous component lacks histologic chondroid features. DDCS accounts for approximately 11% of all chondrosarcoma diagnoses and is associated with a poor prognosis [3]. Grimer et al. documented that the majority of DDCS cases were located in the femur, humerus, and pelvis, followed by the axial skeleton [4]. Chondrosarcoma of the hand and foot is a rare occurrence and DDCS is an even exceedingly rare tumor of the distal extremities, with only four case reports documented in the literature so far [5-8].

## Case presentation

**Figure 1:**
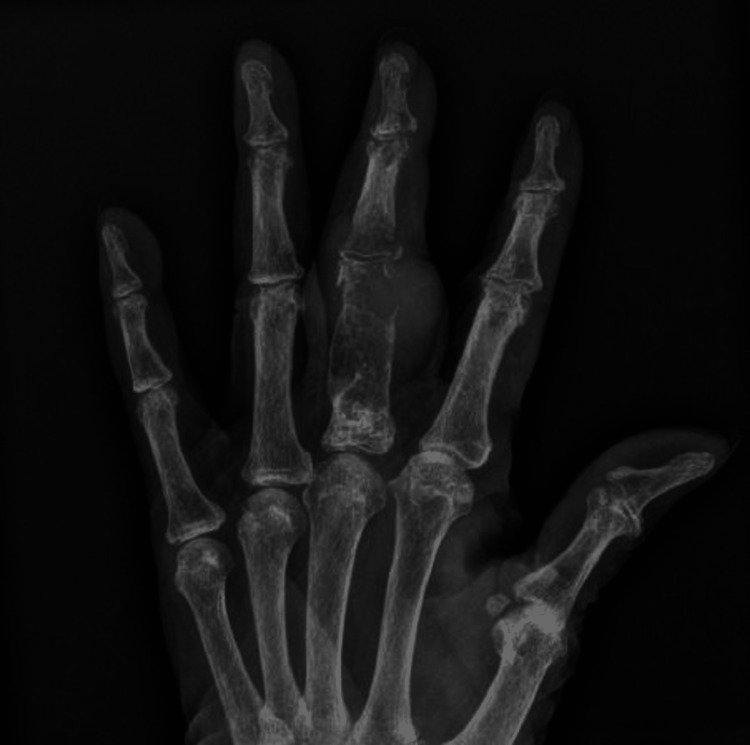
X-ray of the hand showing an expansile lytic lesion with a pathologic fracture of the third proximal phalanx

A 91-year-old female with a past history of diabetes mellitus with neuropathy, hypertension, hypercholesterolemia, and bilateral osteoarthritis of the knees presented with a rapidly enlarging mass on the proximal phalanx of the right middle finger. The mass had been initially noted four months prior when it had been small and clinically thought to be a trigger finger. X-ray imaging showed a pathologic fracture in the neck of the third proximal phalanx with an underlying mildly expansile lytic lesion showing features of a chondroid lesion (Figure 1).

**Figure 2:**
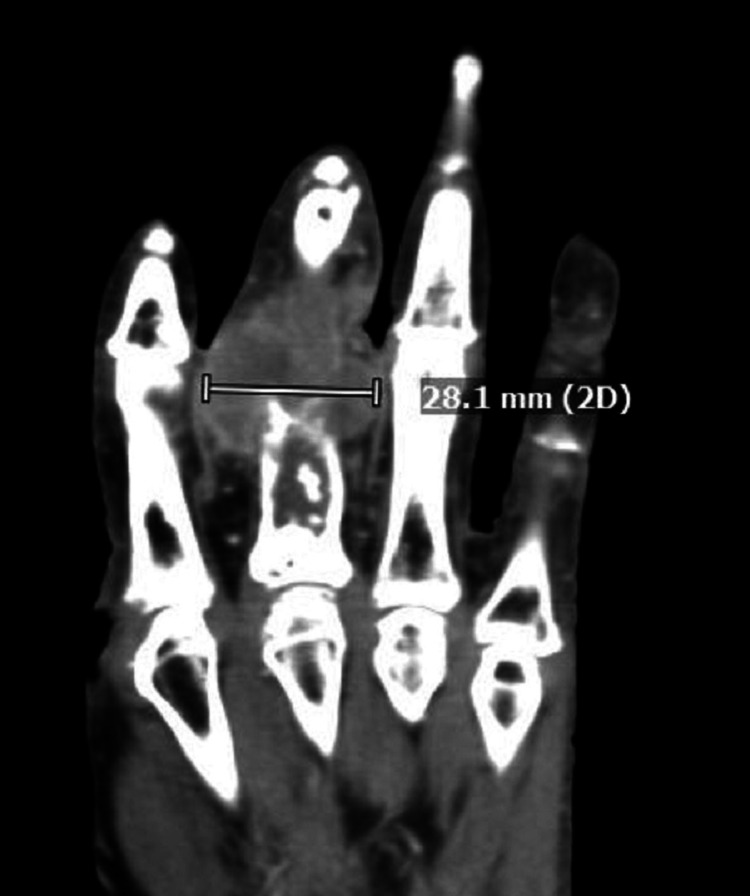
CT scan showing pathologic fracture in the distal aspect of the third proximal phalanx by tumor with chondroid features and extra-osseous soft tissue extension CT: computed tomography

A CT scan further characterized the pathologic fracture in the distal aspect of the third proximal phalanx, demonstrating cortical destruction by a tumor with a chondroid signal and soft tissue extension (Figure 2).

**Figure 3:**
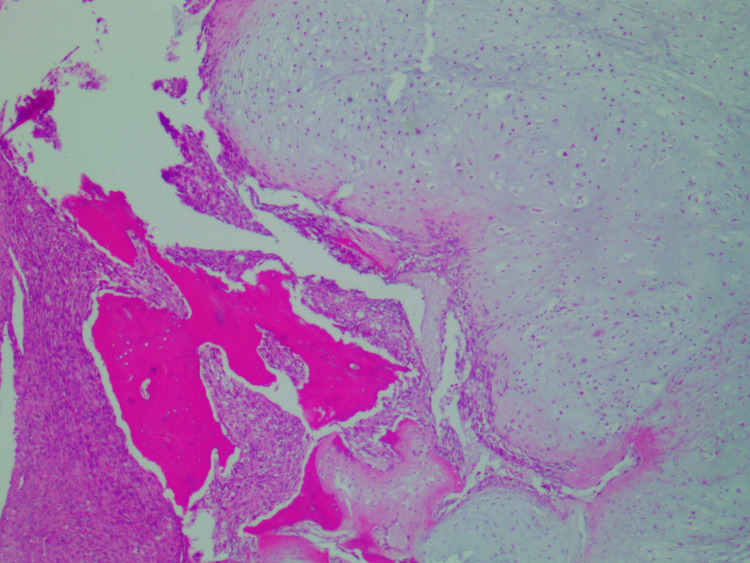
Low-grade chondrosarcoma with adjacent high-grade undifferentiated sarcoma (H&E stain, 200x)

**Figure 4:**
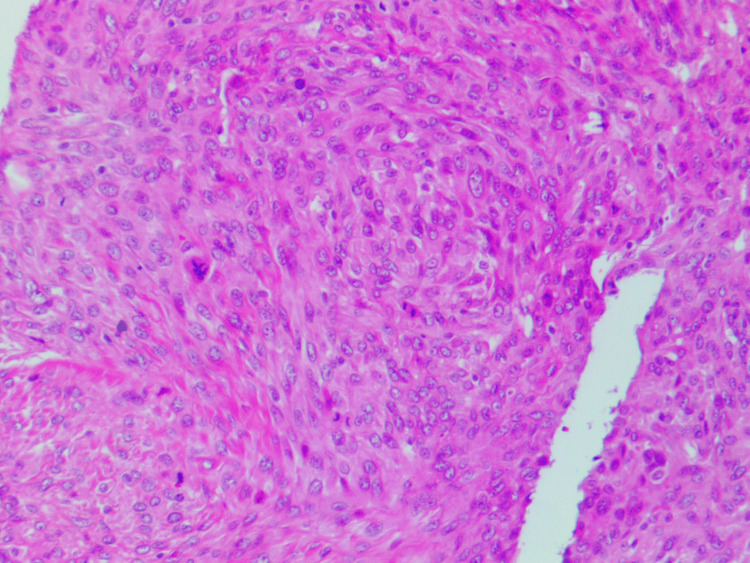
Mitotically active dedifferentiated high-grade undifferentiated sarcoma (H&E stain, 100x)

Erosion along the volar aspect of the base of the third proximal phalanx concerning for a transarticular spread was noted. There were enhancing soft tissue components in and around the fracture site measuring approximately 2.9 x 2.8 x 2.9 cm, demonstrating mixed hyperdense and hypodense signal components. Amputation of the finger was performed and the pathologic examination showed a 3.5 x 3.0 x 2.8-cm mass involving the proximal phalanx, extending to the proximal interphalangeal joint and surrounding soft tissue. The cut surface of the mass was solid and chondroid with a juxtaposed fleshy tan component, replacing the marrow cavity of the proximal phalanx. Microscopic evaluation revealed dedifferentiated chondrosarcoma involving the marrow cavity, proximal interphalangeal joint, and surrounding soft tissue (Figure 3). The chondrosarcoma component was of low grade (grade 1) and well-demarcated from juxtaposed high-grade undifferentiated spindle cell sarcoma. The low-grade chondrosarcoma component infiltrated the marrow, and disrupted the cortical bone with focal slight infiltration in the finger soft tissue, in contrast to the dedifferentiated component that extensively infiltrated the soft tissue. The low-grade chondrosarcoma displayed low cellularity of neoplastic chondrocytes with scant eosinophilic or vacuolated cytoplasm within poorly defined lacunae. The tumor cells, in other areas, were bipolar or stellate-shaped in the myxoid matrix. Cytologic atypia and nuclear pleomorphism were mild with scattered binucleated tumor cells. The dedifferentiated tumor component was characterized by densely cellular fascicles or whorls of the high-grade spindle to ovoid tumor cells with eosinophilic to amphophilic cytoplasm. Nuclear pleomorphism was moderate to marked with brisk mitoses (focally up to 70/10-HPF) and abnormal figures (Figure 4). No histologic features of chondroid differentiation or tumor necrosis were present and collagen in the background was minimal in the dedifferentiated tumor component. Vascular invasion was present and the margins were negative.

Next-generation sequencing showed isocitrate dehydrogenase 2 (*IDH2*) (p.R172S, c.516G>T), *TERT* (c.-146C>T), and *TP53* (c.559+1G>A) mutations. The tumor was equivocal for microsatellite instability and the tumor mutation burden was low. A follow-up CT scan of the chest and a two-month follow-up whole-body positron emission tomography scan showed bilateral innumerable pulmonary nodules. The largest and most active nodule measured up to 2.2 cm. Due to the advanced age of the patient and multiple pulmonary metastases, she was given palliative treatment and was alive at the six-month follow-up.

## Discussion

While chondrosarcoma is the second most common primary sarcoma of the bone overall, it is the most common primary sarcoma of the bone in patients over 50 years of age [1]. However, DDCS is a rare entity, constituting only 1-2% of all primary bone tumors [9]. DDCS is a highly malignant neoplasm and histologically includes two different components. The first component is a well-differentiated cartilage tumor, which is most often a low-grade chondrosarcoma or an enchondroma-like appearance. The second component generally is a high-grade sarcoma lacking cartilaginous features by histology. Both components of the tumor share several similar genetic markers, leading to the conclusion that they are likely derivatives of the same cell line [10]. In our case, the cartilage component was of low grade with adjacent high-grade undifferentiated sarcoma.

Chondrosarcoma of the hand and foot is a rare occurrence, while DDCS affecting the bones of distal extremities is even rarer. In a large series of 35 cases of chondrosarcoma involving only the hand, one case of DDCS was identified and it was excluded from the study [11]. In another case series (23 cases) also looking into chondrosarcoma of the small bones of the hand, one case of DDCS was again excluded [12]. Dedifferentiated cartilaginous tumor of the distal extremities is an exceedingly rare event, with only four detailed case reports documented in the literature, which are as follows: (1) a 66-year-old male with primary DDCS of the proximal phalanx of the left thumb. The patient developed pulmonary metastasis at six months and died of the disease nine months after amputation [5]. (2) An 82-year-old male with primary DDCS of the fifth metacarpal. The patient had an amputation and had a local recurrence after six weeks [6]. (3) A 91-year-old male with primary DDCS of the proximal phalanx of the third toe. He was alive with no evidence of disease 45 months after amputation [7]. (4) A 49-year-old female with secondary DDCS of the proximal phalanx of the fourth finger following a previous diagnosis of enchondroma. She had pulmonary metastases and was alive with the disease at 12 months [8].

Diagnosing DDCS on a limited biopsy is extremely difficult, as the diagnosis depends on recognizing two separate histologic components, and the chance of missing one of the components in a small biopsy is high. Moreover, it is well-established that enchondromas of the hands and feet display greater cellularity and nuclear atypia compared to other body sites, making diagnosing low-grade chondrosarcoma difficult [5].

Conventional chondrosarcoma of the phalanges of the hands and feet is locally aggressive with low metastatic potential [11,13]. Per Bovée et al., only two out of 112 (1.8%) phalangeal chondrosarcoma metastasized [11]. Acral DDCS occurs rarely and cases involving the small bones of the hands and feet are even rarer, making location-based prognosis difficult to ascertain [7]. In our case, the prognosis was dismal as there was a vascular invasion and the CT scan showed multiple pulmonary metastases.

Both benign and malignant central and periosteal cartilage tumors frequently harbor *IDH1/2* mutations and therefore cannot be used as a marker for malignancy. However, it is useful in differentiating chondroblastic osteosarcoma from chondrosarcoma (high-grade or dedifferentiated) as, in most series, chondroblastic osteosar­comas lack* IDH* mutations [14]. Mutant* IDH* chondrosarcomas are metabolically more active tumors when compared to *IDH* non-mutant chondrosarcomas [15]. In our case, an *IDH2* (p.R172S, c.516G>T) mutation was identified.

Zhang et al. found that *TERT* promoter mutations were present in as many as 45% of chondrosarcomas and were significantly associated with high tumor grade, metastasis, and disease-related mortality.*TERT* promoter mutations were also significantly associated with a more aggressive clinical course, including signs of transformation, early metastasis, or a late but aggressive metastasis pattern [16]. In our case, the *TERT* promoter (c.-146C>T) mutation was also identified.

Dedifferentiated and mesenchymal chondrosarcomas have a higher incidence of *TP53* mutations. Overexpression (indicating missense mutations) or alteration in *TP53* is correlated with high histologic grade, presence of metastasis or local recurrence, and reduced overall survival [17]. In our case, a *TP53* (c.559+1G>A) mutation was also identified.

## Conclusions

Acral DDCS is a very rare entity. Diagnosing DDCS on limited biopsy is challenging due to the fact that DDCS contains two components (low-grade chondrosarcoma and another non-cartilage high-grade sarcoma), and the chances of missing one of the components in a limited biopsy are high. Conventional chondrosarcomas of acral bones rarely metastasize, but there is limited literature available on dedifferentiated acral chondrosarcoma, and hence all acral chondrosarcomas including DDCS should be investigated and closely followed up for metastasis. DDCS, in general, has an increased rate of metastasis, particularly from the dedifferentiated tumor component, and has an unfavorable prognosis. Although, identifying *IDH* 1/2 mutations do not distinguish between benign and malignant cartilage tumors, they are useful in differentiating chondroblastic osteosarcoma from chondrosarcoma (high-grade or dedifferentiated). *TERT* promoter mutations or alteration in *TP53* correlate with an increased risk of local recurrence, metastasis, and reduced overall survival.
